# Determinants of Cognitive Performance Among Ghanaian Hypertensive Patients: A Cross‐Sectional Study

**DOI:** 10.1002/hsr2.71100

**Published:** 2025-07-22

**Authors:** Solomon Gyabaah, Samuel Nguah Blay, Shadrack Osei Asibey, Bruce Ovbiagele, Fred Stephen Sarfo

**Affiliations:** ^1^ Komfo Anokye Teaching Hospital Kumasi Ghana; ^2^ Kwame Nkrumah University of Science and Technology Kumasi Ghana; ^3^ University of California San Francisco California USA

**Keywords:** cognitive performance, determinant, Ghanaian, hypertension

## Abstract

**Background:**

Cognitive decline is one of the most deleterious consequences of hypertension. Hypertension is rife in sub‐Saharan Africa, where control of blood pressure is abysmally poor.

**Objective:**

This study is aimed at assessing the determinants of cognitive performance among Ghanaian hypertensive patients.

**Methods:**

This was a cross‐sectional study conducted at a single district hospital among hypertensives aged ≥ 18 years. Global cognitive performance is assessed using the Montreal Cognitive Assessment (MOCA). A multivariable linear regression analysis was performed, and a beta coefficient was computed to identify factors independently associated with the MOCA score.

**Results:**

We enrolled 214 adults living with hypertension; the mean (SD) age was 64 (13.4) years, and 168 (78.5%) were females. Factors, with their adjusted beta coefficients (95% CI), independently associated with cognitive performance were age, −0.10 (−0.16, −0.04), *p* < 0.001; female sex, −2.3 (−4.2, −0.35), *p* = 0.021, secondary and tertiary‐level educational attainment +5.2 (3.5, 6.9), *p* < 0.001 and +4.1 (0.49, 7.7), *p* = 0.026 respectively. In sensitivity unadjusted analyses, body mass index (BMI), with a beta coefficient of +0.18(0.08, 0.29), *p* < 0.001, was associated with MOCA score in females. Among the male participants, a history of heart failure, −11 (−15, −5.9), *p* < 0.001, history of stroke −11 (−15, −5.9), *p* < 0.001, BMI −0.31 (−0.49, −0.13), *p* = 0.002 and uncontrolled hypertension −3.5 (−6.9, −0.15), *p* = 0.047 were associated with MOCA score.

**Conclusion:**

Increasing age and female sex are associated with poorer global cognitive performance, while higher educational attainment is associated with good global cognitive performance among Ghanaians living with hypertension.

## 1 Introduction

An estimated 1.28 billion adults worldwide aged 30–79 years have hypertension, with more than half living in low‐ and middle‐income countries [1]. The pooled prevalence of hypertension in Africa is estimated to be 57% among older adults [2]. In Ghana, Atibila et al., in their systematic review and meta‐analysis, found one in every three individual adults has hypertension [3]. Chronic exposure to high blood pressure leads to changes in the structure and function of blood vessels, leading to decreased cerebral blood flow, small vessel disease, and white matter changes [4]. Hypertension also causes endothelial dysfunction, increasing the risk of microinfarction and other cerebrovascular events among these patients [5]. These alterations contribute to cognitive impairment, affecting executive functions, attention, numerical ability, processing speed, as well as memory [4, 6].

In the Coronary Artery Risk Development in Young Adults (CARDIA) study, it was demonstrated that a resting higher systolic blood pressure from young adulthood is associated with worse cognitive performance as early as midlife [7]. In a systematic review and meta‐analysis by De Heus et al., high blood pressure variability was associated with an increased risk of dementia and cognitive impairment compared to mean blood pressure [8]. That is, blood pressure fluctuations are associated with cognitive impairment regardless of the mean blood pressure. Visit to visit blood pressure variability or day‐to‐day blood pressure variability is an independent predictor of cognitive performance [9]. Hypertension has been shown to significantly impair cognitive function among people living with Human Immunodeficiency Virus (HIV) [10]. Identification and control of hypertension is considered an important step towards population‐level reduction in the global burden of cognitive impairment and dementia [11]. However, while mid‐life hypertension increases the relative risk of lifetime dementia by 20%–54%, the use of antihypertensive therapy is associated with a more modest reduction in the risk of dementia, with a recent meta‐analysis of trials reporting 7%–21% relative risk reduction [12, 13]. Given the high and rising burden of hypertension in sub‐Saharan Africa, the relatively younger age of incident hypertension, abysmally poor control rates of blood pressure on the continent, and a paucity of studies justify an evaluation of the cognitive performance of patients living with hypertension. This study, therefore, sought to assess the determinants of cognitive performance among a cross‐section of Ghanaian hypertensive patients.

## 2 Methods

### 2.1 Study Design and Study Site

This was a cross‐sectional study conducted at the Manhyia district hospital, a primary‐level healthcare facility in Kumasi. Kumasi is the second largest city in the middle belt of Ghana, with an estimated population of 3.4 million in 2020. The Manhyia district hospital has a vibrant adult hypertension clinic run by two nonspecialist medical doctors and four nurses with 800 registrants who visit the clinic every 2 months for antihypertensive medication refills and clinical assessments. Most of the patients who attend the hypertension clinic are registered with the National Health Insurance Scheme with access to subsidized antihypertensive medications and basic laboratory investigations, including blood urea and creatinine, fasting blood glucose, and lipid profile. The classes of antihypertensive medications available on the essential medicine list include ACE inhibitors, angiotensin receptor blockers, beta‐blockers, calcium channel blockers, and thiazide diuretics. The study received ethical approval from the Committee on Human Research Publication and Ethics of the Kwame Nkrumah University of Science and Technology (CHRPE/AP/016/20).

### 2.2 Study Participants

Participants were eligible if they were 18 years or older with a known diagnosis of hypertension presenting for routine care at the general outpatient clinic. Participants were excluded if they had a hypertensive emergency. Informed consent was obtained from all study participants.

### 2.3 Data Collection

All eligible participants provided informed consent before enrollment into the study. With the help of trained research assistants, data on demographic information (age, sex, place of residence, educational status, and monthly income) and lifestyle behaviors (alcohol use, cigarette smoking, level of physical activities, and dietary behavior) were collected through interviews and responses using questionnaires. Individuals were classified as physically active if they were regularly involved in moderate exercise or strenuous exercise for 4 or more hours per week. Smoking status was categorized into ever smoked or never smoked. Vegetable and fruit intake were assessed based on the number of daily servings per week. Information on the duration of hypertension and diabetes diagnosis, and current medication lists were also taken. The duration of hypertension was noted and using the 14‐item version of the Hill–Bone compliance to high blood pressure therapy scale, we assessed the participant's compliance with hypertension treatment [14]. The history of stroke was self‐reported by the participant if they had ever experienced a sudden onset of weakness or sensory loss on one side of the body, sudden loss of vision, or sudden loss of speech. Heart failure was self‐reported if the participant had ever experienced shortness of breath on exertion, lying down, and swelling of both feet. The history of diabetes mellitus was also self‐reported. Blood pressure measurements were performed following a standardized operating procedure. Anthropometric assessments performed by study nurses included measurement of weight and height for body mass index (BMI) derivation as well as waist circumference. Blood pressure variability was assessed by calculating the standard deviation of 10 blood pressure measurements of consecutive 2 monthly visits to the health facility as part of routine follow‐up.

### 2.4 Cognitive Performance

The cognitive performance of the patients was assessed using the Montreal Cognitive Assessment (MOCA) tool [15] by trained research assistants who were blinded to the goals of the study. MoCA is a screening instrument to detect mild cognitive impairment, which was developed by Nasreddine et al. [15]. Most often, it takes about 10–15 min to administer this tool. It is scored from 0–30. The higher your score, the better your cognition [15]. The MOCA consists of several cognitive domains, which include;

- Memory assessment by a short‐term memory recall task (5 points);
- Visuospatial ability testing using a clock‐drawing test (3 points);
- 3‐Dimensional cube copy (1 point);
- Executive function testing using a trail‐making test, (TMT 1 point);
- A phonemic fluency task (1 point);
- 2‐Item verbal abstraction task (2 points);
- Attention, concentration, and working memory are assessed using a sustained attention task (1 point), a serial subtraction task (3 points), and digits forward and backward tasks (1 point each);
- Language is tested using a 3‐item confrontation naming task with low‐familiarity animals (lion, camel, rhinoceros; 3 points);
- Repetition of 2 syntactically complex sentences (2 points);
- Orientation in time and place was also tested (6 points).

The words for the short‐term memory recall task and the TMT‐B were replaced by Ghanaian words and the semantic fluency task was replaced by a phonemic fluency task.

*Outcome measurement:* Cognitive performance of hypertensive patients using MOCA score.

### 2.5 Statistical Analysis

We compared demographic and clinical characteristics of hypertensive patients according to their MOCA score, divided into upper, middle, and lower terciles. The means or medians of continuous variables were compared using either the Analysis of Variance (ANOVA) or the Kruskal–Wallis test. Proportions were compared using a *χ* ^2^ test. A multivariable linear regression analysis was performed and a beta coefficient was computed to identify factors independently associated with MOCA score. An exploratory sensitivity analysis by gender was performed for males and females separately to identify factors associated with MOCA scores without adjustment for confounders due to limited sample size. Variables were included in the multivariate analyses if they met a *p*‐value cutoff of < 0.05 in univariate unadjusted regression analysis. In all analyses, two‐tailed *p* < 0.05 were considered statistically significant. Statistical analysis was performed using R Statistical Software Ver 4.1.1.

## 3 Results

### 3.1 Comparison of Demographic and Clinical Characteristics of Patients by MOCA Score Terciles

We enrolled 214 adults living with hypertension. The overall mean MOCA for all participants was 18 ± 5.9. The median (range) MoCA score for the upper tercile was 25 (22–30), the middle tercile was 19 (17–21), and that of the lower tercile was 12 (1–15). The mean age was 58.9 years for those in the upper tercile of the MOCA score, 64.9 years for those in the middle tercile, and 67.6 years for those in the lower tercile of the MOCA score, *p* < 0.01. There was a significant difference in the proportional distribution of MOCA score in terciles by sex, with more females 70 (89.7%) in the lower tercile of the MOCA score than males 8 (10.3%). Furthermore, the level of education trended significantly (*p* < 0.001) with the MOCA score terciles. Individuals who had no education 58 (74.4%), were more likely to have a lower MOCA score than individuals with primary 10 (12.8%) who were also likely to have a lower MOCA score compared to individuals with secondary 9 (11.5%) and tertiary 1 (1.3%). Also, there was a significant difference (*p *= 0.021 and *p*= 0.025) in the number of antidiabetics and the use of metformin, respectively, among those with comorbid diabetes mellitus by their MOCA score. However, there were no statistically significant differences in MOCA score terciles in terms of income, smoking and alcohol history, salt added to food, physical activity, history of diabetes, stroke, and heart failure, the duration of hypertension, blood pressure variability, and antihypertensive medications (Table 1).

**Table 1:** Comparison of demographic and clinical characteristics of patients by MOCA score.

Characteristic	MOCA score			*p* value^b^
	Upper tercile, *N* = 67^a^	Middle tercile, *N* = 69^a^	Lower tercile, *N* = 78^a^	
Median MOCA score (range)	25 (22–30)	19 (17–21)	12 (1–16)	
Age in years	58.9 (11.6)	64.9 (12.0)	67.6 (14.8)	**< 0.001**
Sex				**< 0.001**
Male	23 (34.3)	14 (20.3)	8 (10.3)	
Female	44 (65.7)	55 (79.7)	70 (89.7)	
Residence				0.65
Rural	7 (10.6)	3 (4.3)	15 (19.2)	
Peri urban	5 (7.6)	7 (10.1)	6 (7.7)	
Urban	54 (81.8)	59 (85.5)	57 (73.1)	
Missing	1	0	0	
Educational level				**< 0.001**
None	10 (14.9)	31 (46.3)	58 (74.4)	
Primary	9 (13.4)	5 (7.5)	10 (12.8)	
Secondary	43 (64.2)	27 (40.3)	9 (11.5)	
Tertiary	5 (7.5)	4 (6.0)	1 (1.3)	
Missing	0	2	0	
Income				0.71
< 100 GHc	5 (7.5)	2 (2.9)	5 (6.4)	
100–500 GHc	18 (26.9)	19 (27.5)	27 (34.6)	
500–1000 GHc	21 (31.3)	23 (33.3)	25 (32.1)	
> 1000 GHc	12 (17.9)	7 (10.1)	2 (2.6)	
Unknown	11 (16.4)	18 (26.1)	19 (24.4)	
Diabetes mellitus	33 (50.0)	33 (47.8)	33 (43.4)	0.43
Missing	1	0	2	
Ever smoked	7 (10.4)	7 (10.1)	5 (6.4)	0.38
Alcohol intake	4 (6.0)	3 (4.3)	2 (2.6)	0.31
Salt added to food	26 (39.4)	24 (34.8)	33 (42.9)	0.64
Missing	1	0	1	
Physical activity	46 (68.7)	42 (60.9)	44 (56.4)	0.13
Daily minutes of exercise	19.6 (18.7)	13.5 (14.2)	14.5 (17.3)	0.07
Missing	0	4	3	
Days of fruit per week	3.2 (2.3)	3.2 (2.1)	2.8 (2.0)	0.29
Daily fruit servings	1.4 (0.9)	1.2 (0.8)	1.4 (1.2)	0.94
Missing	0	1	0	
Days/week vegetables	3.5 (2.5)	3.7 (2.4)	3.6 (2.4)	0.77
Missing	0	0	1	
Daily vegetable servings	1.7 (1.4)	1.3 (0.7)	1.7 (1.2)	0.92
Missing	0	1	2	
Heart failure	4 (6.2)	3 (4.3)	6 (7.9)	0.64
Missing	2	0	2	
Stroke	4 (6.3)	3 (4.5)	6 (8.0)	0.65
Missing	3	2	3	
BMI	31.7 (11.3)	29.8 (5.5)	29.6 (7.8)	0.15
Missing	4	6	5	
Waist circumference	98.4 (21.4)	98.4 (16.9)	99.7 (22.9)	0.68
Missing	4	4	8	
Duration of hypertension in years	7.3 (7.2)	8.8 (7.6)	7.8 (6.4)	0.72
Missing	0	0	2	
ACE‐inhibitors	10 (14.9)	10 (14.5)	5 (6.4)	0.10
ARB use	46 (68.7)	40 (58.0)	55 (70.5)	0.76
Beta‐blockers use	1 (1.5)	1 (1.4)	5 (6.4)	0.09
Calcium channel blockers	57 (85.1)	62 (89.9)	62 (79.5)	0.32
Diuretics	13 (19.4)	13 (18.8)	15 (19.2)	0.98
Methyldopa	6 (9.0)	2 (2.9)	2 (2.6)	0.08
Hydralazine	2 (3.0)	2 (2.9)	0 (0.0)	0.18
No. of antidiabetics	0.6 (0.8)	0.4 (0.7)	0.3 (0.6)	**0.021**
Metformin	26 (38.8)	19 (27.5)	17 (21.8)	**0.025**
Sulphonylurea	11 (16.4)	7 (10.1)	6 (7.7)	0.10
Thiazolidinedione	2 (3.0)	3 (4.3)	5 (6.4)	0.33
Insulin	2 (3.0)	1 (1.4)	1 (1.3)	0.46
Statin	20 (29.9)	20 (29.0)	21 (26.9)	0.69
Antiplatelet	26 (38.8)	16 (23.2)	29 (37.2)	0.91
No. of antihypertensive	2.0 (0.9)	1.9 (0.7)	1.9 (0.9)	0.18
Hill–Bone score	24.2 (4.3)	22.8 (3.9)	23.5 (5.2)	0.43
Missing	7	4	6	
Systolic variability	14.9 (6.1)	15.4 (7.0)	16.8 (7.6)	0.12
Missing	4	16	8	
Diastolic variability	10.4 (4.5)	10.1 (4.2)	11.7 (5.9)	0.12
Missing	4	16	8	

*Note:* p‐values highlighted were those that attained level of statistical significance of *p* < 0.05.

Abbreviations: ACE = angiotensin converting enzyme, ARB = angiotensin receptor blocker, MOCA = Montreal Cognitive Assessment.

^a^ Mean (SD); *n* (%).

^b^ Ordinal cumulative link model; *χ* ^2^ trend test.

### 3.2 Factors Associated With Cognitive Performance Assessed Using the MOCA Score

In a multivariable linear regression model, increasing age was significantly associated with a lower MOCA score with an adjusted beta coefficient (95% CI) of −0.10 (−0.16, −0.04), *p* < 0.001. Also, being a female was significantly associated with a lower MOCA score with an adjusted beta coefficient (95% CI) of −2.3 (−4.2, −0.35), *p*= 0.021. Secondary and tertiary education were significantly associated with a higher MOCA score with an adjusted beta coefficient (95% CI) of +5.2 (3.5, 6.9), *p* < 0.001 and +4.1 (0.49, 7.7), *p*= 0.026, respectively. There was no significant association between MOCA score and blood pressure control, Hill and Bone score, history of diabetes mellitus, stroke, heart failure, body mass index, and the duration of hypertension (Table 2).

**Table 2:** Factors associated with poor cognitive performance.

Characteristic	Crude				Adjusted		
	*N*	Beta	95% CI^a^	*p* value	Beta	95% CI^a^	*p* value
Age in years	214	−0.13	−0.18, −0.07	**< 0.001**	−0.10	−0.16, −0.04	**< 0.001**
Sex	214						
Male		—	—		—	—	
Female		−3.2	−5.1, −1.3	**0.001**	−2.3	−4.2, −0.35	**0.021**
Educational level	212						
None		—	—		—	—	
Primary		2.5	0.16, 4.8	**0.037**	1.6	−0.73, 4.0	0.18
Secondary		6.3	4.7, 7.8	**< 0.001**	5.2	3.5, 6.9	**< 0.001**
Tertiary		6.6	3.2, 10	**< 0.001**	4.1	0.49, 7.7	**0.026**
Diabetes mellitus	211						
No		—	—				
Yes		0.63	−0.96, 2.2	0.44			
Ever smoked	214						
No		—	—				
Yes		1.8	−1.0, 4.6	0.21			
Alcohol intake	214						
No		—	—				
Yes		2.7	−1.3, 6.6	0.19			
Physical activity	214						
No		—	—		—	—	
Yes		1.6	−0.07, 3.2	0.06	0.45	−1.3, 2.2	0.61
Daily minutes of exercise	207	0.04	−0.01, 0.09	0.09	0.01	−0.04, 0.06	0.76
Days of fruit per week	214	0.36	−0.01, 0.74	0.06	0.27	−0.07, 0.62	0.12
Heart failure	210						
No		—	—				
Yes		−2.4	−5.8, 0.86	0.15			
Stroke	206						
No		—	—				
Yes		−2.4	−5.8, 0.87	0.15			
BMI	199	0.08	−0.02, 0.18	0.12			
Duration of hypertension in years	212	0.01	−0.11, 0.12	0.90			
No. of antihypertensive	214	0.34	−0.60, 1.3	0.48			
Hill–Bone score	197	0.01	−0.18, 0.19	0.95			
Systolic blood pressure	186	−0.02	−0.09, 0.04	0.45			
Diastolic blood pressure	186	0.07	−0.03, 0.16	0.16			
Controlled blood pressure	186						
Controlled		—	—				
Uncontrolled		−0.59	−2.4, 1.2	0.53			

Abbreviation: BMI = body mass index.

^a^ CI = confidence interval.

### 3.3 Sensitivity Analysis by Gender

In a sensitivity analysis for males and females separately, increasing age was significantly associated with a lower MOCA score in both males and females with a beta coefficient (95% CI) of 0.14 (−0.27, −0.01), *p* = 0.041 and −0.15 (−0.21, −0.09), *p* < 0.001 respectively. For the female participants, secondary and tertiary education was associated with a higher MOCA score with a beta coefficient (95% CI) of +5.6 (3.9, 7.3), *p* < 0.001 and +13 (7.5, 18), *p* < 0.001, respectively. There was no significant association between the MOCA score and females with primary education. However, for the male participants, primary education was significantly associated with higher MOCA scores with a beta coefficient (95% CI) of +8.6 (3.6, 14), *p* = 0.002. Also, secondary education was associated with a higher MOCA score among the male participants with a beta coefficient of +8.3 (4.8, 12), *p* < 0.001. There was no significant association between male participants with tertiary education and MOCA score, with a beta coefficient (95% CI) of +3.2 (−1.5, 8.0), *p* = 0.193.

Among the female participants other factors such as days of fruit consumption per week with a beta coefficient of +0.45 (0.04, 0.86), *p*= 0.032, and body mass index (BMI) with beta coefficient (95% CI) of, *p*< 0.001 were positively associated the MOCA score. Among the male participants, a history of heart failure with beta coefficient of −11 (−15, −5.9), *p* < 0.001, history of stroke with beta coefficient of −11 (−15, −5.9), *p* < 0.001, BMI with a beta coefficient of −0.31 (−0.49, −0.13), *p*= 0.002 and uncontrolled hypertension with a beta coefficient of −3.5 (−6.9, −0.15), *p*= 0.047 were associated with lower MOCA score. Also, the number of antihypertensives with a beta coefficient of +2.5 (1.0, 4.0), *p*= 0.002 was associated with a higher MOCA score (Table 3).

**Table 3:** Sensitivity analysis by gender.

Characteristic	Female				Male			
	*N*	Beta	95% CI^a^	*p* value	*N*	Beta	95% CI^a^	*p* value
Age in years	169	−0.15	−0.21, −0.09	**< 0.001**	45	−0.14	−0.27, −0.01	**0.041**
Educational level	168				44			
None		—	—			—	—	
Primary		0.99	−1.5, 3.5	0.45		8.6	3.6, 14	**0.002**
Secondary		5.6	3.9, 7.3	**< 0.001**		8.3	4.8, 12	**< 0.001**
Tertiary		13	7.5, 18	**< 0.001**		3.2	−1.5, 8.0	0.19
Diabetes mellitus	166				45			
No		—	—			—	—	
Yes		0.88	−0.91, 2.7	0.34		−1.6	−4.9, 1.6	0.32
Ever smoked	169				45			
No		—	—			—	—	
Yes		−1.0	−5.0, 2.9	0.61		2.7	−1.1, 6.5	0.17
Alcohol intake	169				45			
No		—	—			—	—	
Yes		2.7	−1.7, 7.2	0.23		2.4	−5.4, 10	0.55
Physical activity	169				45			
No		—	—			—	—	
Yes		1.6	−0.27, 3.4	0.09		2.6	−0.51, 5.8	0.11
Daily minutes of exercise	163	0.04	−0.02, 0.09	0.17	44	0.07	−0.02, 0.16	0.12
Days of fruit per week	169	0.45	0.04, 0.86	**0.032**	45	0.49	−0.34, 1.3	0.25
Heart failure	166				44			
No		—	—			—	—	
Yes		0.55	−3.4, 4.5	0.79		−11	−15, −5.9	**< 0.001**
Stroke	162				44			
No		—	—			—	—	
Yes		0.57	−3.4, 4.6	0.78		−11	−15, −5.9	**< 0.001**
BMI	158	0.18	0.08, 0.29	**< 0.001**	41	−0.31	−0.49, −0.13	**0.002**
Duration of hypertension in years	167	−0.02	−0.15, 0.11	0.74	45	0.06	−0.14, 0.27	0.55
No. of antihypertensive	169	−0.21	−1.3, 0.92	0.711	45	2.5	1.0, 4.0	**0.002**
Hill–Bone score	153	−0.05	−0.26, 0.15	0.610	44	0.19	−0.17, 0.54	0.311
Systolic blood pressure	144	−0.02	−0.09, 0.05	0.531	42	−0.05	−0.16, 0.07	0.424
Diastolic blood pressure	144	0.07	−0.05, 0.18	0.238	42	0.06	−0.09, 0.21	0.455
Controlled blood pressure	144				42			
Controlled		—	—			—	—	
Uncontrolled		0.03	−2.0, 2.1	0.974		−3.5	−6.9, −0.15	**0.047**

Abbreviation: BMI = body mass index.

^a^ CI = confidence interval.

## 4 Discussion

Hypertension is an important risk factor for cognitive impairment and decline globally. In this cross‐sectional study among Ghanaians living with hypertension, we found increasing age and female sex were independently associated with poorer cognitive performance, while higher levels of educational attainment were associated with better cognitive performance. An unadjusted sensitivity analysis among males identified potentially modifiable adverse factors associated with lower cognitive performance, including uncontrolled blood pressure, history of heart failure, history of stroke, and increasing BMI, while a higher number of antihypertensives was associated with better cognitive performance among males. Among females, we found that regular consumption of fruits and higher BMI were associated with better cognitive performance.

Age is an important non‐modifiable risk factors that generally influence cognitive performance. There is evidence showing that the human brain undergoes structural changes during normal ageing with loss of synaptic complexity and reduced white and grey matter volume [16]. The volume and/or the weight of the human brain reduces at a rate of 0.2% per annum after age 35% and 5% per decade after age 40, with an accelerated decline after age 70 [17, 18, 19]. Executive function, abstraction, mental flexibility, visuoperceptual judgment, and the ability to perceive spatial orientation decline with age [16]. In addition, there is a loss of vascular elasticity and compliance, leading to stiffer vessels that are less resilient as one ages. These age‐related microvascular and macrovascular changes may impair blood supply to the brain. These declines, importantly, may be accelerated in patients with hypertension. Persistent exposure to pulsatile stress of hypertension promotes microvascular damage that results in brain atrophy, and loss of cortical connections, thus affecting cognitive performance [20].

One important issue that is commonly raised on the concept of cognitive performance is the role our environment plays in our behavior and cognitive function. The concept of cognitive reserve refers to the ubiquitous finding that, during later life, individuals higher in experiential resources, such as education and knowledge, exhibit higher levels of cognitive function compared to individuals with low experiential resources [21]. Educational attainment has been shown to reflect a more effective use of brain networks or cognitive paradigms [22]. Our data showed that educational level influences cognitive performance among hypertensive patients, with better performance occurring in those patients with higher education levels. Muela et al. found higher educational level to be associated with good cognitive performance among hypertensives [22]. In Tanzania, Pallangyo et al. showed that hypertensive patients with low education had an over threefold chance of having cognitive impairment [23].

Regarding sex, it has been shown that females have higher cognitive decline compared to their male counterparts, with increased prevalence of Alzheimer's disease among women, partly because women tend to live longer than men [24, 25]. Other studies attribute the difference in cognitive performance to a decline in estrogen levels found in females in their menopausal age [26]. In a systematic review of 26,088 individuals, it was shown that females compared to males have a faster decline in global cognition and executive function, and this difference persisted after adjusting for the influence of age, race, education, and mean blood pressure [25]. In a cross‐sectional study involving 3690 adults with hypertension, Farron et al. showed that female sex is independently associated with poor cognitive performance [27]. Our study buttresses these gender‐related differences in cognitive performance in a low‐income setting.

There have been unclear findings on the influence of body mass index (BMI) on cognitive performance. In a cross‐sectional study of 1545 participants, Feinkohl et al. demonstrated that older people who are obese are at higher risk of cognitive impairment compared with normal weight and overweight individuals which was independent of co‐morbidity such as hypertension or diabetes [28]. However, some studies report the reverse relationship between BMI and cognitive function, indicating the uncertain and ambiguous relationship for their link. In a nationally representative study of the Korean population, Kim et al. showed that obesity was associated with a lower risk of cognitive decline among the mid‐ and old‐age population [29]. Kim and Yeom found a U‐shaped pattern association between BMI and cognitive decline, with both the underweight and the overweight or obese patients experiencing poor cognitive performance, which was heterogeneous by sex or cardiovascular risk [30]. Overall, this study found no association between BMI and cognitive performance. However, in a sensitivity analysis, this study showed that increased body mass index among hypertensive patients is associated with poor cognitive performance among males but better cognitive performance among females.

Antidiabetic medications such as metformin, sodium glucose co‐transporter‐2 inhibitors (SGLT‐2), glucagon‐like peptide‐1 agonist (GLP‐1), and Dipeptidyl peptidase‐4 (DPP‐4) inhibitors have been shown to improve cognitive performance among diabetic patients [31, 32, 33]. They provide a neuroprotective effect on the brain. Ng et al. observed a significant neuroprotective effect of using metformin in both cross‐sectional and longitudinal analyses [34]. Metformin has been shown to improve processing speed, memory, verbal and visual learning, and also enhances the functional connectivity of the dorsolateral prefrontal cortex [35]. In this study, we also found that the use of antidiabetic medications and metformin was associated with good cognitive performance. However, in a systemic review and meta‐analysis by Malazy et al., there was no significant relationship between metformin therapy and cognitive performance [36].

Poor cognitive performance is common in patients with heart failure, with a reported prevalence of 20%–80% [37], and is associated with poor self‐care, more frequent rehospitalisations, and increased mortality [38]. Also, stroke is an independent risk factor for the development of cognitive impairment and dementia. A review of studies involving about 300,000 participants in 12 countries suggested that the prevalence of post‐stroke cognitive impairment ranges from 20%–80%, varying among countries, races, and diagnostic criteria [39]. Among Ghanaian stroke survivors, Sarfo et al. showed nearly half had cognitive impairment [40]. In this study, we found among the male participants that a history of heart failure, stroke, and uncontrolled blood pressure was associated with poor cognitive performance. However, these factors did not influence cognitive performance among the female participants in this study. In this sensitivity analysis, an adjusted beta coefficient was not done due to the smaller sample size of the male participants. SPRINT MIND Trial showed that intensive BP control does not have a harmful effect on cognitive function and cerebral perfusion [41]. Low diastolic blood pressure levels should not be a block to intensive blood pressure control [41].

### 4.1 Limitations

This is a single‐center study; hence, findings may not be generalizable. Furthermore, causal inferences between factors identified to be associated with cognitive performance cannot be made due to the cross‐sectional design of the study. There was no normative control population to define cut‐offs for cognitive impairment. Furthermore, a limited sample size precluded the conduct of adjusted analyses to identify factors associated with cognitive performance by sex. Also, the study did not consider the psychological stress or the personality traits of the participants. Further studies are therefore needed to address some of these limitations. Indeed, future prospective studies are urgently needed in resource‐limited settings to better characterize the factors associated with cognitive trajectories of adults living with hypertension due to the high burden of hypertension and its deleterious impact on neurocognitive and cerebrovascular health.

## 5 Conclusion

Increasing age and female sex are associated with poorer global cognitive performance, while higher educational attainment is associated with good global cognitive performance among this sample of Ghanaians living with hypertension. Older males with higher BMI, heart failure, and stroke may need more rigorous vascular risk management to improve their cognitive performance and quality of life.

## Author Contributions

**Solomon Gyabaah:** conceptualization, data curation, formal analysis, investigation, methodology, project administration, validation, visualization, writing – original draft, writing – review and editing. **Samuel Nguah Blay:** data curation, formal analysis, writing – original draft, writing – review and editing. **Shadrack Osei Asibey:** investigation, methodology, project administration, writing – original draft, writing – review and editing. **Bruce Ovbiagele:** funding acquisition, investigation, methodology, resources, supervision, validation, visualization, writing – original draft, writing – review and editing. **Fred Stephen Sarfo:** conceptualization, funding acquisition, investigation, methodology, project administration, resources, supervision, validation, visualization, writing – original draft, writing – review and editing.

## Ethics Statement

The study was approved by the Committee on Human Research Publication and Ethics of the Kwame Nkrumah University of Science and Technology.

## Consent

Written informed consent was obtained from the patients.

## Conflicts of Interest

The authors declare no conflicts of interest.

## Transparency Statement

The corresponding author, Fred Stephen Sarfo, affirms that this manuscript is an honest, accurate, and transparent account of the study being reported; that no important aspects of the study have been omitted; and that any discrepancies from the study as planned (and, if relevant, registered) have been explained.

## Data Availability

Data is available upon reasonable request to the corresponding author.
